# Editorial Note: Iron-Ascorbate-Mediated Lipid Peroxidation Causes Epigenetic Changes in the Antioxidant Defense in Intestinal Epithelial Cells: Impact on Inflammation

**DOI:** 10.1371/journal.pone.0289575

**Published:** 2023-07-31

**Authors:** The PLOS ONE Editors

During the discussion of the Expression of Concern decision on this article [1, 2], PLOS asked the authors to replicate experiments presented in Fig 3, 4, and 6, to clarify whether the article’s results are reliable. Due to the time needed to conduct and review these experiments, the replication data were not included with the Expression of Concern notice [2] and are being provided in this follow-up notice. Information on the methodology and the repeat experiment data obtained are presented in the S1 File below. The results of repeat experiments and the consistency with other results in this article were assessed by an independent member of the *PLOS ONE* Editorial Board. The board member’s comments and the authors’ point-by-point response are provided in S2 File below.

The board member reviewed the authors’ response in S2 File and commented that the repeat experiment data presented for Fig 3E appear inconsistent with the published results, but also indicated that these data are not critical for the overall conclusions of the study. Furthermore, the board member commented that the fold difference between the published results in Fig 6A-D and the results obtained during the repeat experiments is acceptable as the overall trend observed in the data remains consistent.

Based on our assessment, the new data support the article’s conclusions, but they do not resolve the concerns raised in the Expression of Concern about image data reporting. Therefore, the *PLOS ONE* editors issue this Editorial Note to provide an update to the published Expression of Concern [2], and to share the repeat experiment data and outcome of the editorial assessment of these data.

## Supporting information

S1 File Author response to concerns and results of new experiments. (PDF)

S2 File Reviewer comments and point-by-point responses. (PDF)
