# Supplementary material for: Editorial Note: Iron-Ascorbate-Mediated Lipid Peroxidation Causes Epigenetic Changes in the Antioxidant Defense in Intestinal Epithelial Cells: Impact on Inflammation
Source: PLoS One. 2023 Jul 31;18(7):e0289575. doi: 10.1371/journal.pone.0289575 (PMC10389698; doi:10.1371/journal.pone.0289575)
Supplement: S1 File — (PDF) [file pone.0289575.s001.pdf]

## Materials and Methods

### Cell Culture and Treatments

The human epithelial colorectal adenocarcinoma Caco-2/15 cell line, obtained from Dr. JF Beaulieu (Department of Cellular Biology, Faculty of Medicine, Université de Sherbrooke, Sherbrooke, Quebec, Canada) were grown at 37$^0$C with 5% $CO_2$ in minimal essential medium (MEM) (GIBCO-BRL, Grand Island, NY) containing 1% penicillin-streptomycin, 1% MEM nonessential amino acids (GIBCO-BRL) and supplemented with 10% decomplemented fetal bovine serum (FBS) (Flow, McLean, VA) as described previously. Passage 20 Caco-2/15 cells were seeded at a density of $5.10^5$ cells/well in 6-well plate with flat bottom (Costar, Cambridge, MA), in MEM containing 10% FBS and grown for 2 days. Before exposure to Fe-Asc (200 mM/2 mM) for 6 h at 37$^0$C, Caco-2/15 cells were pre-incubated with 0.25 mM 6-hydroxy-2,5,7,8-tetramethylchromane-2-carboxylic acid (Trolox), with ethanol as a vehicle, for 24 h. Four experimental cell groups were considered: control (with 0.25 mM ethanol as a vehicle), Fe-Asc (to induce OxS), Trolox (as antioxidant) and Trolox+Fe-Asc (to assess the direct contribution of OxS via its neutralisation by the antioxidant). For DNA methylation, Caco-2/15 cells were pre-incubated with 5-aza-2'-deoxycytidine (5- AZA, kindly obtained by Dr R Noel) at a concentration of 10 mM for 24 h , and 5 experimental cell groups were established: control (with ethanol as a vehicle), 5-AZA (to preclude methylation), Fe-Asc+5-AZA (to prevent methylation in the presence of OxS), Trolox+5-AZA (to evaluate the combined effect of antioxidant and demethylating agent), and Fe-Asc+Trolox+5-AZA (to evaluate the combined effect of antioxidant and demethylating agent in the presence of OxS).

### Determination of gene expression by PCR

Amplicons were generated from cDNA using Taq DNA Polymerase (Invitrogen) according to manufacturer's instructions. In brief, 28 cycles of amplification were used with initial denaturation at 94$^0$C for 30 min, subsequent denaturation at 94$^0$C for 45 s, annealing at 58$^0$C for 30 s, and 72$^0$C for 30 s.The primers used for the SOD2 amplification were: sense primer 5'-CGACCTGCCCTACGACTACG-3' and antisense primer 5'- TGACCACCACCATTGAACTT-3'. The primers used for the GPx amplification were: 5'- TTCGCTCTGAGGCACAACC-3' and antisense primer 5'- ACAGGGCTCCAAATGATGAG-3'. GAPDH was used as reference and the primers used were: sense primer 5'-AATCCCATCACCATCTTCCA -3' and antisense primer 5'- TGGACTCCACGACGTACTCA-3'. PCR was performed using the UNO II thermocycler (Biometra). Amplicons were visualized on standard ethidium bromide-stained agarose gels. The number of amplification cycles corresponds to the linear portion of the exponential phase, as determined in preliminary experiments. Fold induction was calculated using GAPDH as a housekeeping gene, and quantification was determined with Image Lab software (Bio-Rad).

### Immunoblotting

To determine the protein expression of cyclooxygenase-2 (COX-2), IkB, nuclear factor-kB (NF-kB), Caco-2/15 cells were lysed in ice-cold RIPA buffer containing 20 mM Tris-HCl (pH 7.5), 150 mM NaCl, 1 mM $Na_2$EDTA,1 mM EGTA, 1% NP-40, 1% deoxycholate, 2.5 mM sodium pyro-phosphate, 1 mM $Na_2VO_4$, 1 µg/ml leupeptin, and 1 mM PMSF. Aliquots of homogenates containing 20 µg total proteins were subjected to 8% SDS-PAGE and electroblotted onto nitrocellulose membranes. The membranes were cut into strips according to the molecular weight to focus on the corresponding protein of interest. These slices were then incubated overnight at 4°C with the specific primary antibodies of Cox-2 (1/1000; Novus, Oakville, ON); NF-κB p65 subunit (1/5,000; Santa Cruz Biotechnology); inhibitor κBα (I-κBα, 1/250, Invitrogen); and β-actin (1/250,000; Sigma-Aldrich) used as an internal control. All the proteins of interest from the same membrane shared the same loading control (β-actin). After incubation with the relative secondary antibody, mouse IgG-POD/rabbit IgG-POD (1/10,000; Roche Diagnostics), immune complexes were revealed using Clarity Max Western ECL substrate (Bio-Rad). Reactive bands were captured using a Chemi-Doc MP Imaging System (Bio-Rad). All data are expressed as the ratio of target protein to β-actin in the same sample.

# Figure 3 (previously published): Effects of oxidative stress on transcription factor NF-κB in Caco-2/15 cells.

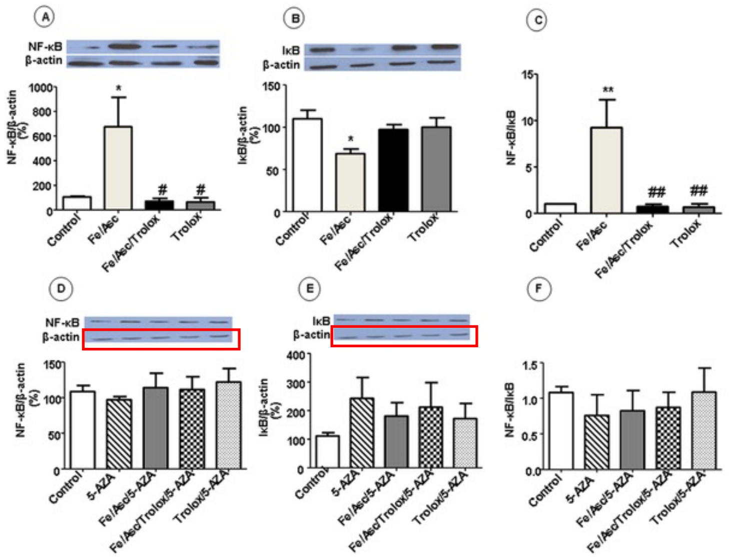


**Concern: The following results appear similar : The Fig 3D NF-κB panel and the Fig 3E IκB panel.**

**Response**: The actin is the same because the ptoteins NF-kB (65 kDA, I-kB (36 kDA) and Actin (42 kDA) were revealed on the same membrane, which is usual in many laboratories (especially when several proteins, related to metabolic pathways, are invesitigated (as illustrated below).

**The experiments have now been repeated and the results are similar to 2013 (see below)**

**Figure 3D & 3E (new experiments-2022): Effects of oxidative stress on transcription factor NF-κB in Caco-2/15 cells.**

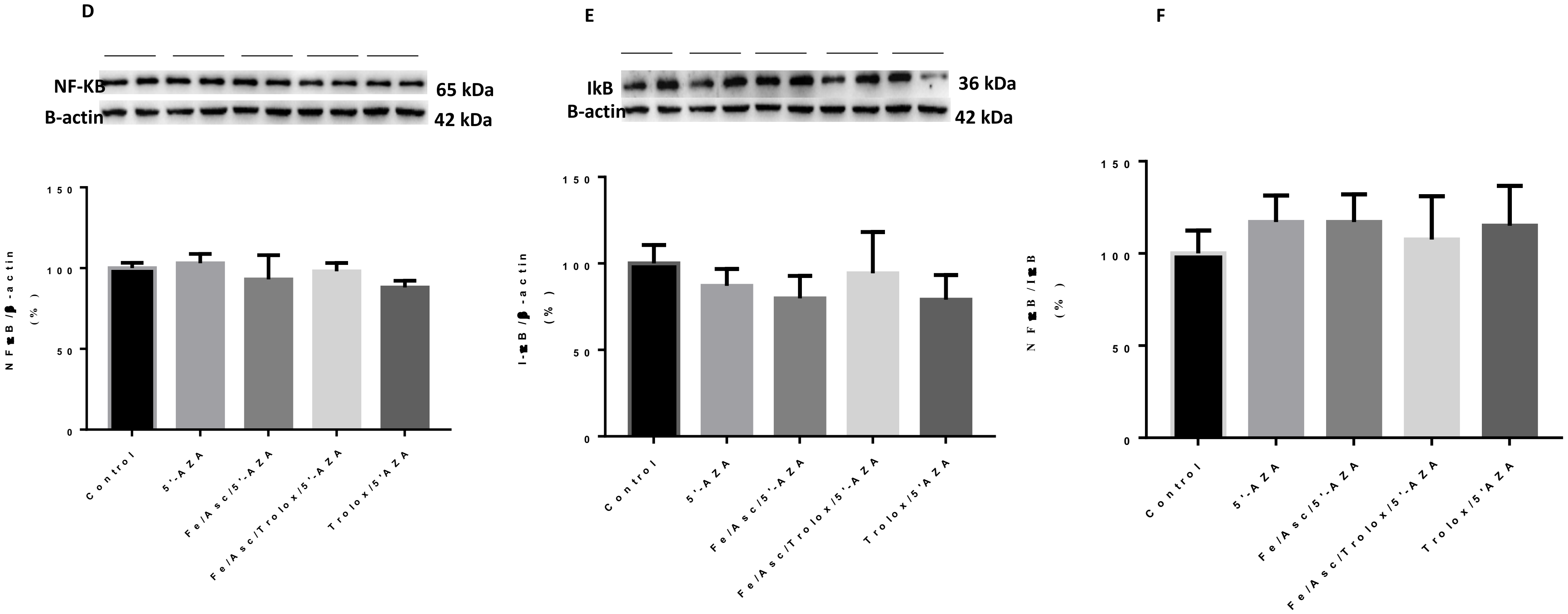


**Figure 3D-3F (2022). Effects of oxidative stress on transcription factor NF-kB in Caco-2/15 cells.** Caco-2/15 cells were first incubated with Trolox (0.25 mM) and/or 5-AZA (10 mM) for 24h. They were then treated with Fe-Asc (200 mM/ 2 mM) for 6 h at $37^0$C. The protein expression of (D) NF-kB and (E) I-kB were determined by western blot as described in Materials and Methods. Importantly, NF-kB (65 kDA, I-kB (36 kDA) and Actin (42 kDA) were loaded on the same gel, revealed on the same membrane, and therefore shared the same β-actin.

Then the (F) NF-kB/IkB ratio was calculated. Results represent the means SEM of 2 independent experiments in duplicates.

## Figure 3D & 3E (new experiments-2022): Effects of oxidative stress on transcription factor NF-κB in Caco-2/15 cells.

### First series of experiments (n=2/Tx)

NF-kB

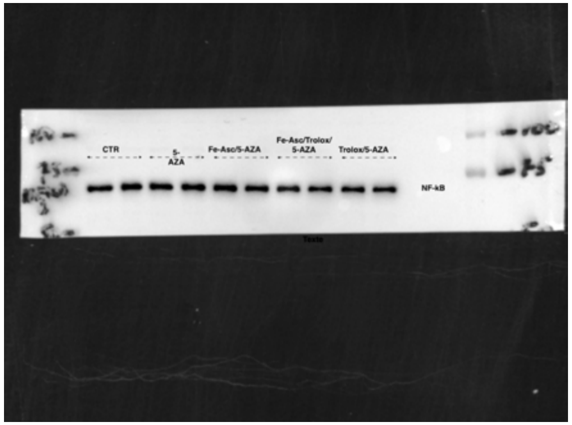

I-kB

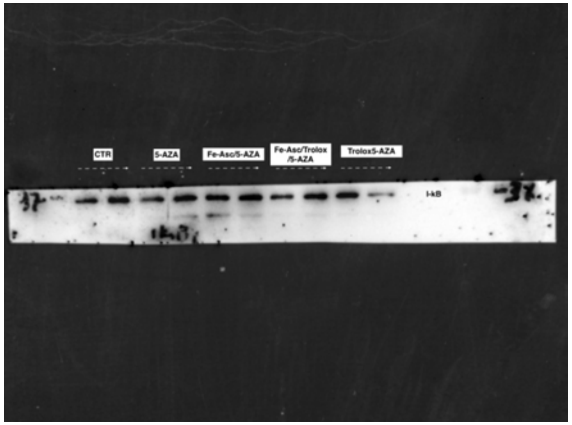

β-actin

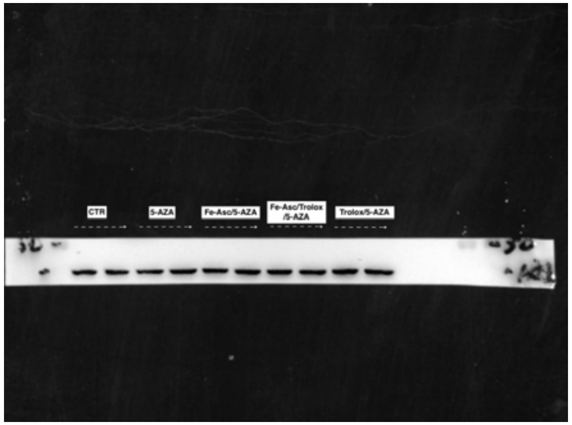

| NFkB_1_Ctrl-5AZA-5AZAFeAsc-Trolox5AZAFeAsc-Trolox5AZA | | | | | | |
|---|---|---|---|---|---|---|
| | NFKB1 | IKB1 | Actine1 | NFKB1/Actine1 | IKB1/Actine1 | NFKB1/IKB1 |
| | Adj. Volume (Int) | Adj. Volume (Int) | Adj. Volume (Int) | | | |
| **C1** | 4438784 | 2561680 | 2894940 | 1,5332905 | 0,884881897 | 1,73276287 |
| **C2** | 4473336 | 3424388 | 2891278 | 1,547182941 | 1,18438559 | 1,30631692 |
| **5'-AZA1** | 4667520 | 2618066 | 2879577 | 1,620904737 | 0,909184231 | 1,7828122 |
| **5'-AZA2** | 5095950 | 3151060 | 3594584 | 1,417674479 | 0,87661326 | 1,6172177 |
| **5'-AZA/FeAsc1** | 5357525 | 2780888 | 3295320 | 1,625798102 | 0,843890123 | 1,92655188 |
| **5'-AZA/FeAsc2** | 4316448 | 3382698 | 3607520 | 1,196513949 | 0,937679625 | 1,27603706 |
| **Trol/5'-AZA/FeAsc1** | 4438380 | 2426996 | 3078900 | 1,441547306 | 0,788267238 | 1,82875456 |
| **Trol/5'-AZA/FeAsc2** | 4599056 | 3642936 | 3247773 | 1,416064485 | 1,121671989 | 1,26245863 |
| **Trol/5'-AZA1** | 4486584 | 3625644 | 3277302 | 1,368987051 | 1,106289259 | 1,2374585 |
| **Trol/5'-AZA2** | 4853332 | 2607628 | 3196875 | 1,518148817 | 0,815680313 | 1,86120566 |

## Figure 3D & 3E (new experiments-2022): Effects of oxidative stress on transcription factor NF-κB in Caco-2/15 cells.

**Second series of experiments (n=2/Tx)**

NF-kB

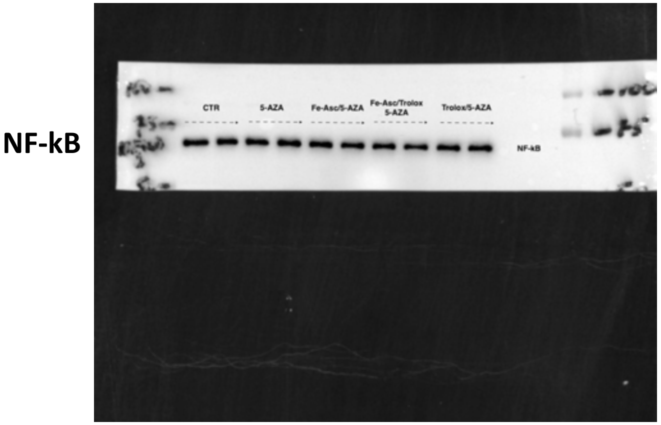

I-kB

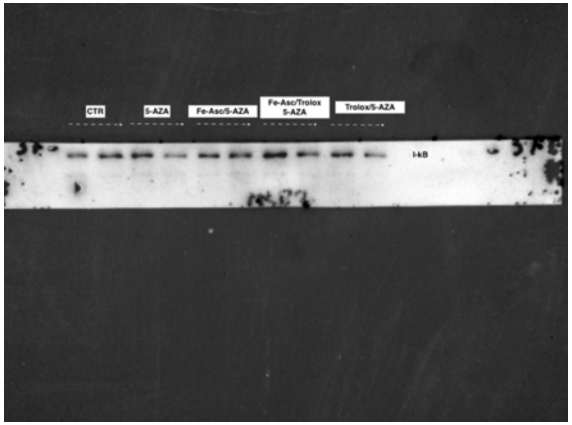

B-actin

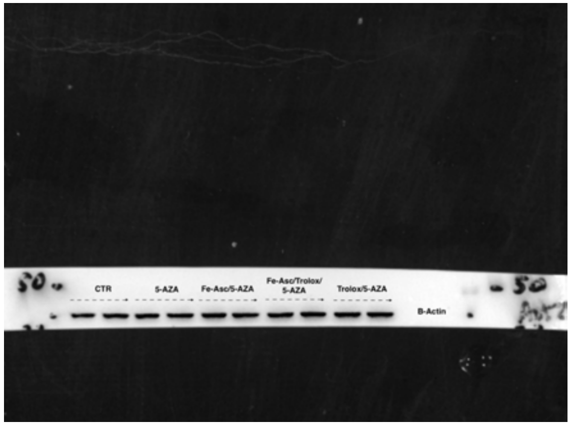

| NFkB_2_Ctrl-5AZA-5AZAFeAsc-TroloxFeAsc5AZA-Trolox5AZA | | | | | | |
|---|---|---|---|---|---|---|
| | NFKB2 | IKB2 | Actine2 | NFKB2/Actine2 | IKB2/Actine2 | NFKB2/IKB2 |
| | Adj. Volume (Int) | Adj. Volume (Int) | Adj. Volume (Int) | | | |
| C3 | 3849607 | 1874442 | 2453548 | 1,568996001 | 0,763972011 | 2,05373492 |
| C4 | 4531389 | 2372260 | 3147456 | 1,439698919 | 0,753707121 | 1,91015698 |
| 5'-AZA3 | 4621114 | 2093608 | 2881080 | 1,60395199 | 0,726674719 | 2,20724892 |
| 5'-AZA4 | 5029176 | 1889090 | 3043560 | 1,652399164 | 0,62068433 | 2,66222149 |
| 5'-AZA/FeAsc3 | 4362816 | 1707552 | 3766320 | 1,158376346 | 0,453374116 | 2,55501209 |
| 5'-AZA/FeAsc4 | 4882436 | 1918994 | 2900175 | 1,68349703 | 0,66168214 | 2,54426851 |
| Trol/5'-AZA/FeAsc3 | 5670648 | 3159376 | 3382512 | 1,676460571 | 0,934032459 | 1,79486329 |
| Trol/5'-AZA/FeAsc4 | 4861674 | 1797158 | 3399500 | 1,430114429 | 0,528653626 | 2,70520121 |
| Trol/5'-AZA3 | 4639845 | 2117390 | 3683783 | 1,25953266 | 0,574786843 | 2,19130392 |
| Trol/5'-AZA4 | 4396418 | 1516216 | 3493881 | 1,25831933 | 0,433963263 | 2,89959874 |

# Strips of the two membranes after cutting

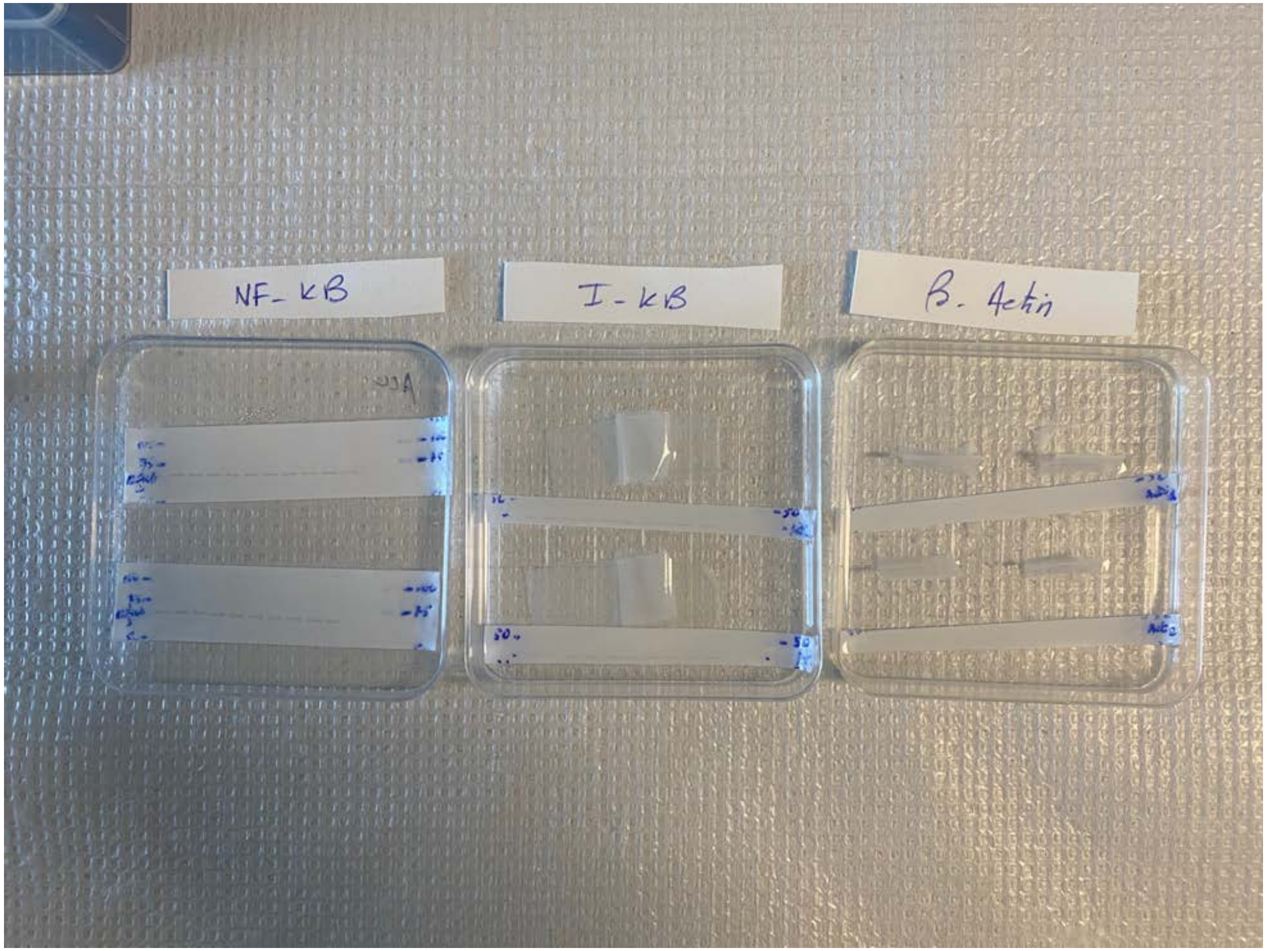


Typical Ponceau Red stained membrane after transfer

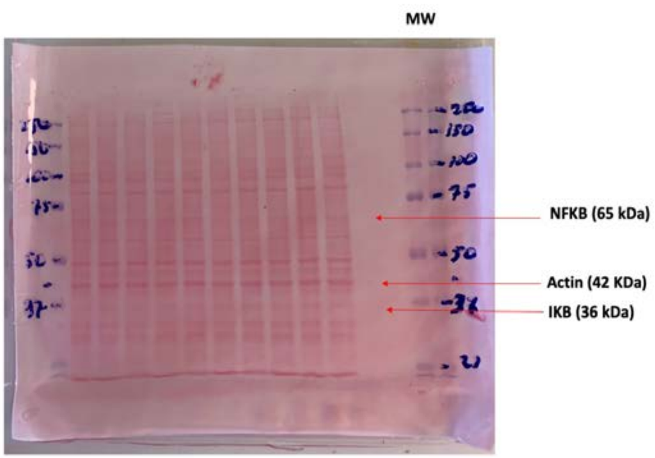

## Figure 4 (previously published): . Effects of oxidative stress on inflammatory markers in Caco-2/15 cells.

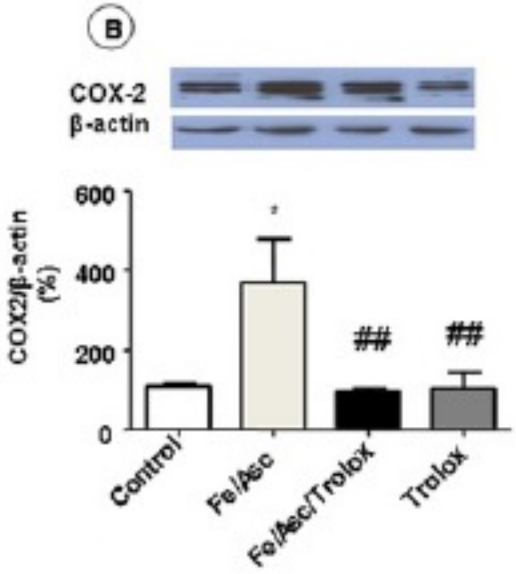


**Concern: When levels are adjusted to visualize background, there appear to be vertical irregularities suggestive of splice lines in the Fig 4B COX-2 panel, between lanes 1–2 and lanes 3–4.**

**Response**: Often, the gel are representative of three differents experiments performed in duplicates or triplicates. The representation of one spot requires splicing to reduce the load of the representative Figure and prevents it from being overloaded

**The experiments have now been repeated, and the results are similar to those in 2013 (see below).**

**Figure 4B (new experiments-2022): Effects of oxidative stress on inflammatory markers in Caco-2/15 cells.**

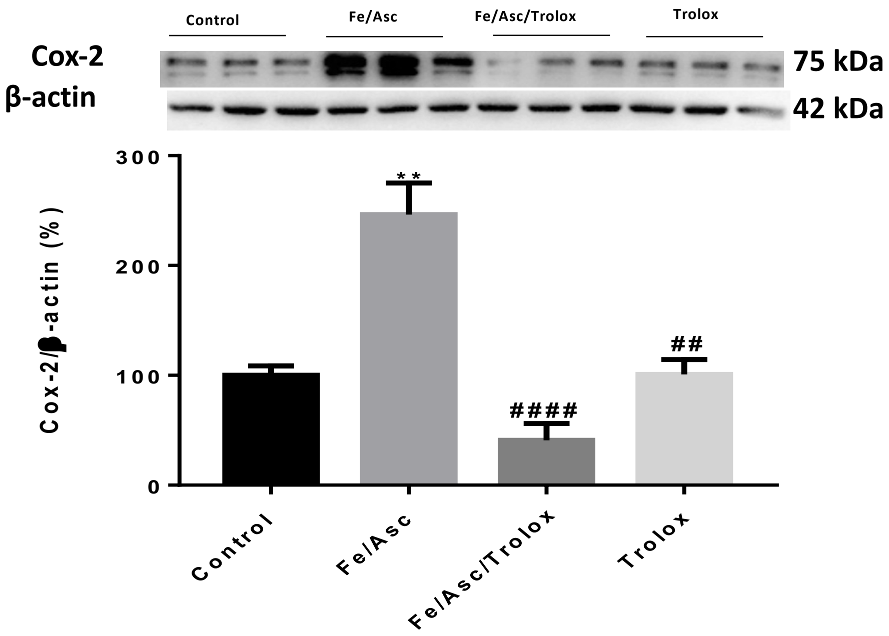


Prior to incubation with Fe/Asc (200 mM/2 mM) for 6 h, cells were challenged with Trolox (0.25 mM) for 24 h at $37^{0}$C. The protein expression of cyclooxygenase 2 (COX-2) was determined by western blot as described in Materials and Methods. Results represent the means SEM of one experiment carried in triplicates. **P<0.01 vs. controls; ##P<0.01, ####P< 0.001 vs. Fe/Asc.

# Figure 4B (new experiments-2022): Effects of oxidative stress on inflammatory markers in Caco-2/15 cells.

COX2

β-actin

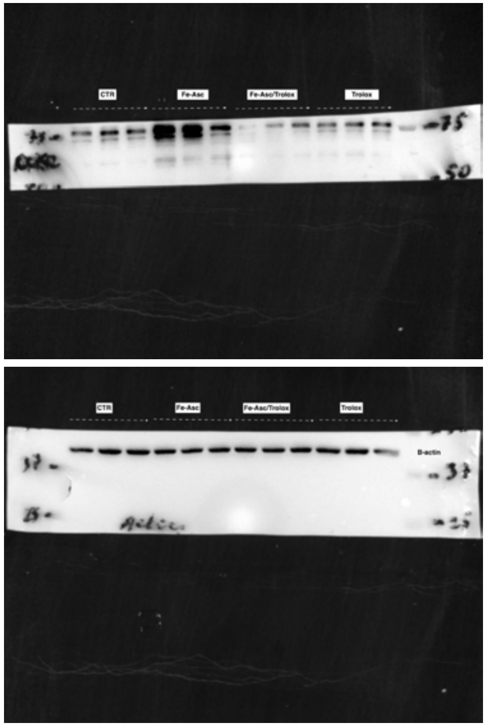


| Cox2-FeAsc | | | |
|---|---|---|---|
| | Cox-2 | Actine | Cox-2/Actine |
| | Adj. Volume (Int) | Adj. Volume (Int) | |
| C1 | 1886997 | 2912602 | 0,647873276 |
| C2 | 3235806 | 4096400 | 0,789914559 |
| C3 | 2754150 | 4070858 | 0,676552707 |
| FeAsc1 | 6778107 | 3730386 | 1,816998831 |
| FeAsc2 | 6682074 | 3363910 | 1,986400944 |
| FeAsc3 | 4997581 | 3560898 | 1,403460869 |
| Tro/FeAsc1 | 470921 | 3520110 | 0,133780194 |
| Tro/FeAsc2 | 827631 | 3504534 | 0,236160071 |
| Tro/FeAsc3 | 1857912 | 3772626 | 0,492471822 |
| Trolox | 2307879 | 3937472 | 0,586132168 |
| Trolox | 2729832 | 4036802 | 0,676236288 |
| Trolox | 3001767 | 3448730 | 0,870397799 |

Strips of the membrane after cutting

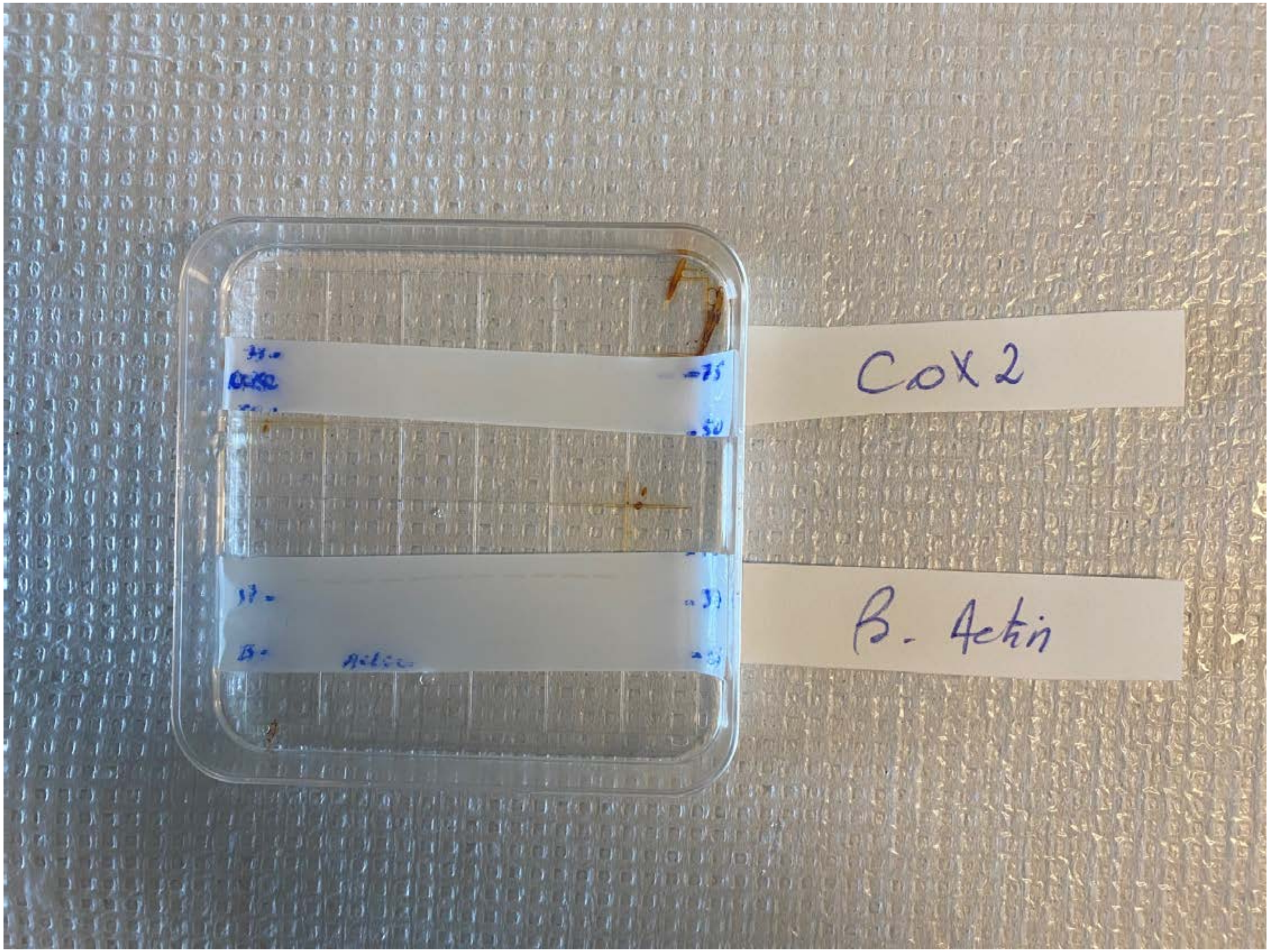

**Figure 6 (previously published): Effects of oxidative stress on antioxidant genes in Caco-2/15 cells.**

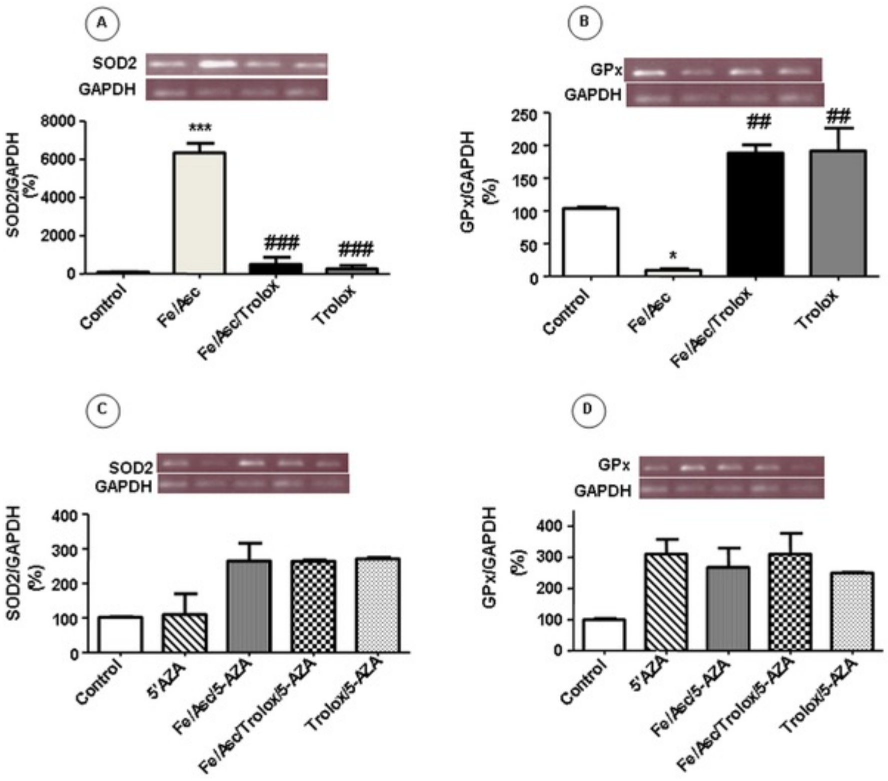


**Concern: Fig 6A GAPDH panel, Fig 6B GAPDH panel, Fig 6C GAPDH panel lanes 1–4, and Fig 6D GAPDH panel lanes 1–4. In addition, lanes 5 of the Fig 6C and 6D GAPDH panels also appear similar.**

- **Fig 6B GPx panel lanes 2–4, and Fig 6D GPx panel lanes 1–3.**
- **Fig 6C SOD2 panel lanes 1–2 and Fig 6D GPx panel lanes 4–5.**

**Reponse**: The experiments regarding Figure 6 have now been repeated to eliminate any possible confusion (see below).

## Figure 6 (new experiments-2022): Effects of oxidative stress on antioxidant genes in Caco-2/15 cells.

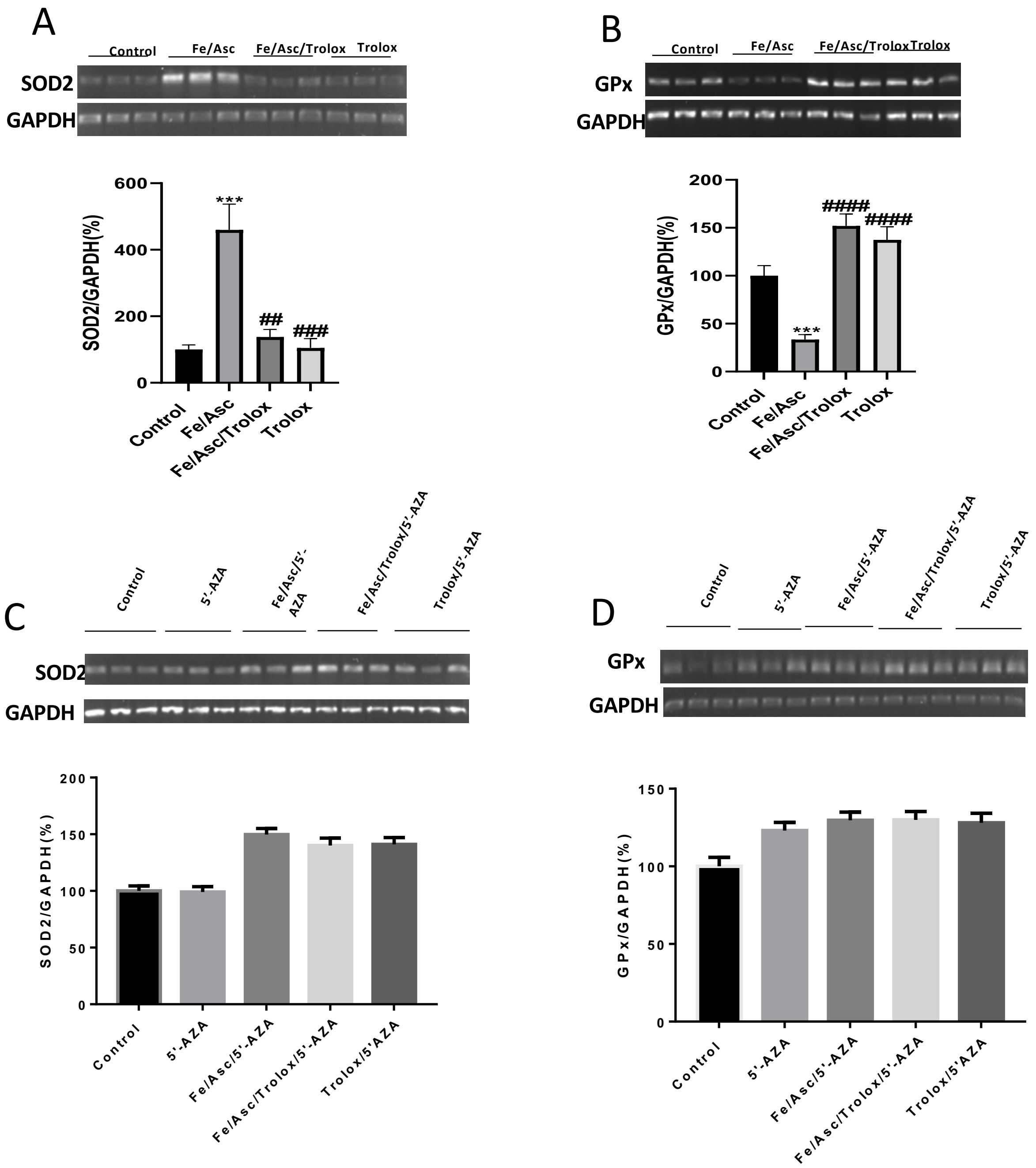


**Figure 6. Effects of oxidative stress on antioxidant genes in Caco-2/15 cells.** Cells were incubated with Fe/Asc (200 mM/2 mM) and Trolox (0.25 mM) for 6 h at 37$^0$C and/or with 5-AZA (10 mM).

Gene expression of SOD2 (A, C) and GPx (B, D) were then evaluated as described in Materials and Methods. Results represent the means SEM of n=3 for one or two independent experiments.

***P<0.001vs. controls; ##P<0.01, ###P<0.001, ####P<0.0001 vs Fe/Asc.

## Figure 6A-B: New Experiments with n=3/treatment

SOD2

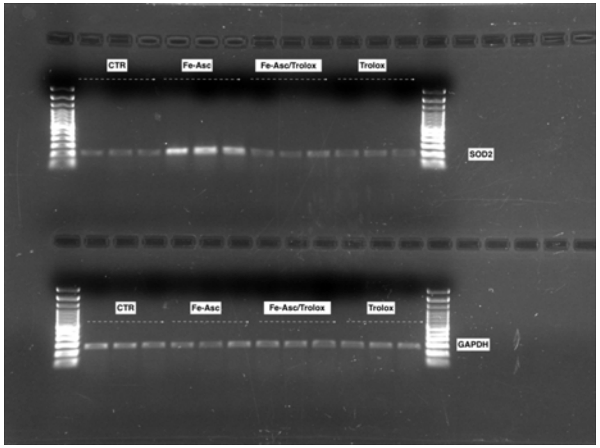


GPX

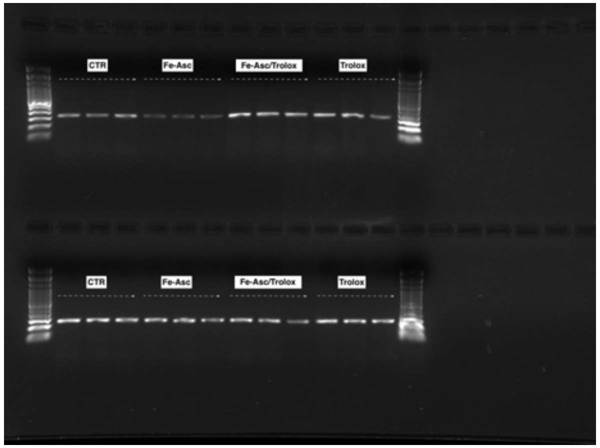


| | SOD2-GAPDH-Ctrl-FeAsc-TrolFeAsc-Trolox | | |
|---|---|---|---|
| | **SOD2** | **GAPDH** | **SOD2/GAPDH** |
| | Adj. Volume (Int) | Adj. Volume (Int) | |
| **C1** | 2473460 | 5282640 | 0,468224221 |
| **C2** | 2436005 | 4519625 | 0,538983876 |
| **C3** | 2467575 | 3792965 | 0,650566246 |
| **FeAsc1** | 7044125 | 3306160 | 2,130606202 |
| **FeAsc2** | 10037605 | 3096330 | 3,24177494 |
| **FeAsc3** | 9536120 | 4236595 | 2,250892521 |
| **Tro/FeAsc1** | 2937330 | 3852585 | 0,762430939 |
| **Tro/FeAsc2** | 2129545 | 3636820 | 0,585551388 |
| **Tro/FeAsc3** | 3320350 | 3551680 | 0,934867443 |
| **Trolox1** | 2464935 | 3302475 | 0,746390207 |
| **Trolox2** | 2620860 | 3761120 | 0,696829668 |
| **Trolox3** | 1120845 | 3823690 | 0,293131765 |

| GPX-GAPDH-Ctrl-FeAsc-TrolFeAsc-Trolox | | | |
|---|---|---|---|
| | **GPx** | **GAPDH** | **GPx/GAPDH** |
| | Adj. Volume (Int) | Adj. Volume (Int) | |
| **C1** | 8156610 | 15739295 | 0,51823223 |
| **C2** | 7895340 | 14264085 | 0,55351184 |
| **C3** | 8821450 | 13317535 | 0,6623936 |
| **FeAsc1** | 2076775 | 13249885 | 0,1567391 |
| **FeAsc2** | 2421865 | 13343385 | 0,18150304 |
| **FeAsc3** | 2298445 | 9494870 | 0,2420723 |
| **Tro/FeAsc1** | 10964500 | 12994355 | 0,84378948 |
| **Tro/FeAsc2** | 9604615 | 11255200 | 0,85334912 |
| **Tro/FeAsc3** | 8013615 | 8546435 | 0,93765588 |
| **Trolox1** | 11621335 | 14683295 | 0,79146642 |
| **Trolox2** | 11742940 | 13248675 | 0,88634826 |
| **Trolox3** | 9192615 | 13033405 | 0,70531185 |

# Figure 6C: New experiments with n=3/Treatment

SOD2 (1)

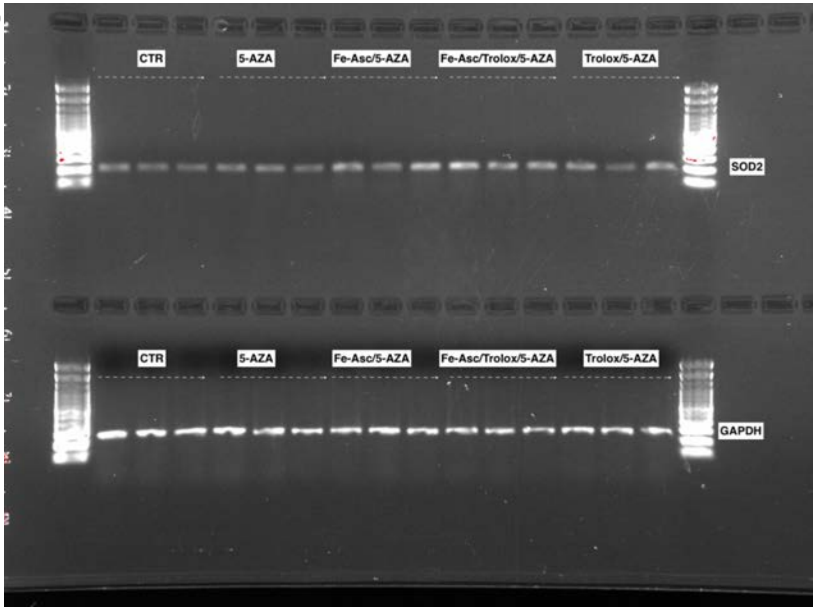


| SOD2-GAPDH-Ctrl-5AZA-5AZAFeAsc-Trol5AZAFeAsc-Trol5AZA | | | |
|---|---|---|---|
| | SOD2 | GAPDH | SOD2/GAPDH |
| | Adj. Volume (Int) | Adj. Volume (Int) | |
| C1 | 18140920 | 29000175 | 0,625545191 |
| C2 | 18775344 | 22061933 | 0,851028965 |
| C3 | 21245784 | 28039440 | 0,75771071 |
| 5'-AZA1 | 15027320 | 18390081 | 0,817142676 |
| 5'-AZA2 | 12807592 | 22404819 | 0,57164452 |
| 5'-AZA3 | 16333464 | 26671725 | 0,612388738 |
| 5'-AZA/FeAsc1 | 19273184 | 22080546 | 0,872858126 |
| 5'-AZA/FeAsc2 | 18717104 | 19519520 | 0,958891612 |
| 5'-AZA/FeAsc3 | 13055504 | 19403598 | 0,672839336 |
| Trol/5'-AZA/FeAsc1 | 21425656 | 26561886 | 0,806631577 |
| Trol/5'-AZA/FeAsc2 | 14896392 | 23105805 | 0,644703441 |
| Trol/5'-AZA/FeAsc3 | 12317832 | 28317429 | 0,434991185 |
| Trol/5'-AZA1 | 10631376 | 23335189 | 0,455594167 |
| Trol/5'-AZA2 | 9505064 | 15982327 | 0,594723409 |
| Trol/5'-AZA3 | 9918152 | 19433823 | 0,510355168 |

SOD2 (2)

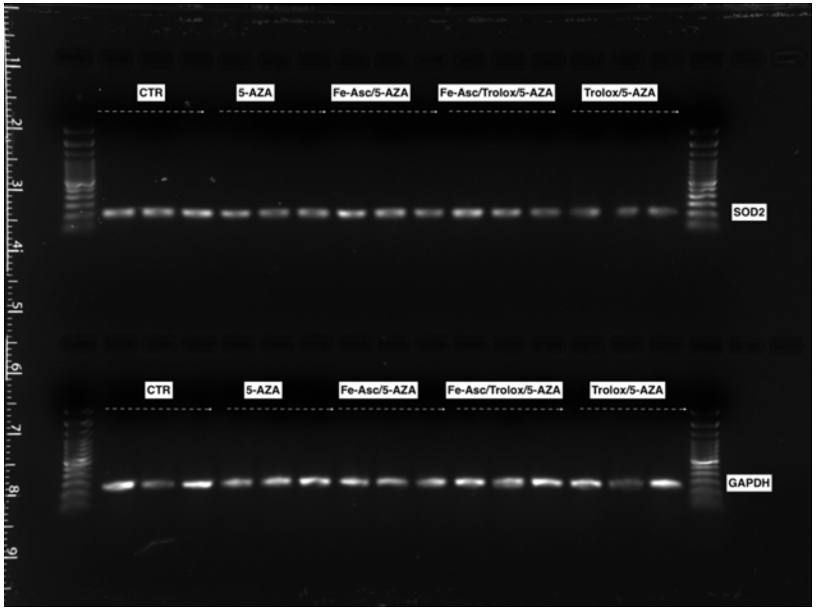


| | SOD2-GAPDH-Ctrl-5AZA-5AZAFeAsc-Trol5AZAFeAsc-Trol5AZA | | |
|---|---|---|---|
| | SOD2 | GAPDH | SOD2/GAPDH |
| | Adj. Volume (Int) | Adj. Volume (Int) | |
| C1 | 3957968 | 13202336 | 0,299793006 |
| C2 | 2700936 | 11251688 | 0,240047182 |
| C3 | 2604784 | 10043488 | 0,259350536 |
| 5'-AZA1 | 3217696 | 12475848 | 0,257914011 |
| 5'-AZA2 | 2884240 | 8756376 | 0,329387409 |
| 5'-AZA3 | 2845192 | 9783952 | 0,290801917 |
| 5'-AZA/FeAsc1 | 4044744 | 8025736 | 0,503971723 |
| 5'-AZA/FeAsc2 | 3976736 | 8208824 | 0,484446493 |
| 5'-AZA/FeAsc3 | 4085800 | 8008960 | 0,510153628 |
| Trol/5'-AZA/FeAsc1 | 4289208 | 8186696 | 0,523924181 |
| Trol/5'-AZA/FeAsc2 | 4184024 | 7528712 | 0,555742337 |
| Trol/5'-AZA/FeAsc3 | 3883808 | 7995056 | 0,48577621 |
| Trol/5'-AZA1 | 4570888 | 8263136 | 0,553166256 |
| Trol/5'-AZA2 | 4078448 | 8084744 | 0,504462232 |
| Trol/5'-AZA3 | 5030888 | 7723464 | 0,651377154 |

# Figure 6D: new experiments with =3/treatment

GPX (1)

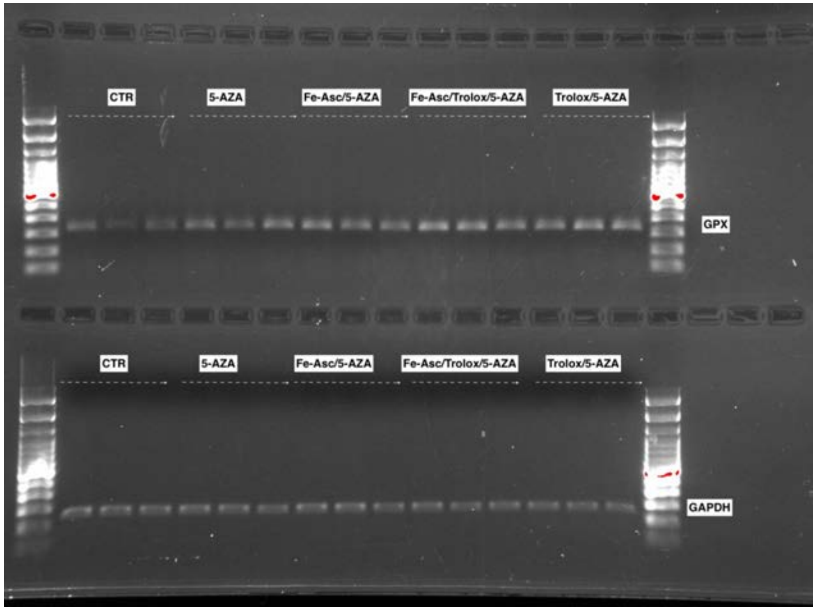


| GPX-GAPDH-Ctrl-5AZA-5AZAFeAsc-Trol5AZAFeAsc-Trol5AZA | | | |
|---|---|---|---|
| | GPx | GAPDH | GPx/GAPDH |
| | Adj. Volume (Int) | Adj. Volume (Int) | |
| C1 | 9902368 | 25562512 | 0,387378517 |
| C2 | 11336024 | 24418992 | 0,464229809 |
| C3 | 7428064 | 21523264 | 0,345117915 |
| 5'-AZA1 | 13700120 | 29544424 | 0,463712544 |
| 5'-AZA2 | 12732664 | 29289792 | 0,434713364 |
| 5'-AZA3 | 10729544 | 26029136 | 0,412212837 |
| 5'-AZA/FeAsc1 | 11321296 | 24940104 | 0,453939406 |
| 5'-AZA/FeAsc2 | 13121248 | 28552050 | 0,459555373 |
| 5'-AZA/FeAsc3 | 12290824 | 26102266 | 0,470871916 |
| Trol/5'-AZA/FeAsc1 | 14102480 | 29127128 | 0,484169946 |
| Trol/5'-AZA/FeAsc2 | 14375928 | 30489060 | 0,471511027 |
| Trol/5'-AZA/FeAsc3 | 12796040 | 27025288 | 0,473483946 |
| Trol/5'-AZA1 | 10311216 | 25307016 | 0,407444955 |
| Trol/5'-AZA2 | 11075168 | 21159096 | 0,523423496 |
| Trol/5'-AZA3 | 11369992 | 27225632 | 0,417620866 |

GPX (2)

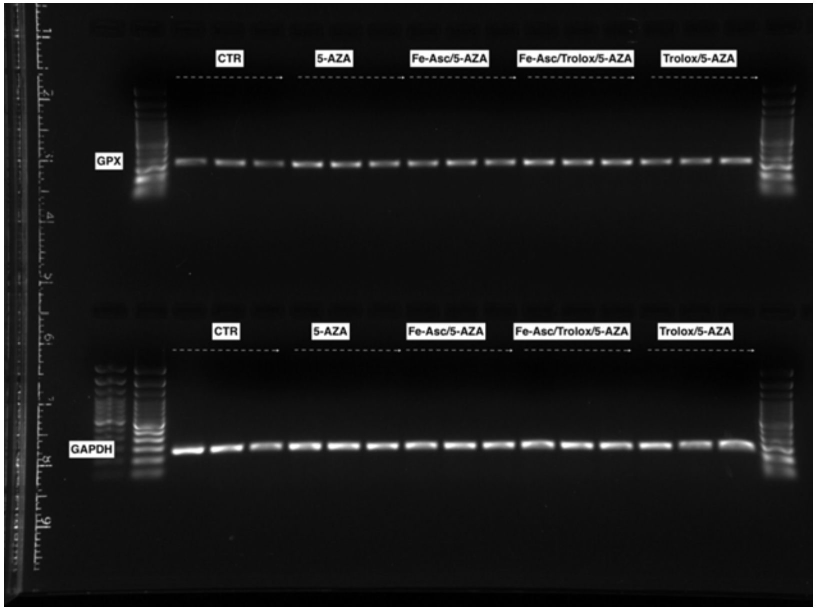


| | GPX-GAPDH-Ctrl-5AZA-5AZAFeAsc-Trol5AZAFeAsc-Trol5AZA | | |
|---|---|---|---|
| | GPx | GAPDH | GPx/GAPDH |
| | Adj. Volume (Int) | Adj. Volume (Int) | |
| C1 | 3281992 | 4226432 | 0,77653964 |
| C2 | 2247608 | 4247608 | 0,52914676 |
| C3 | 2561560 | 3492976 | 0,73334601 |
| 5'-AZA1 | 4078160 | 4501144 | 0,90602745 |
| 5'-AZA2 | 3878488 | 4073864 | 0,9520416 |
| 5'-AZA3 | 3920536 | 4063144 | 0,96490206 |
| 5'-AZA/FeAsc1 | 4025664 | 4531424 | 0,88838829 |
| 5'-AZA/FeAsc2 | 4384488 | 4255712 | 1,03025957 |
| 5'-AZA/FeAsc3 | 4209296 | 4163280 | 1,01105282 |
| Trol/5'-AZA/FeAsc1 | 4298088 | 4439136 | 0,96822625 |
| Trol/5'-AZA/FeAsc2 | 4157072 | 4367376 | 0,9518466 |
| Trol/5'-AZA/FeAsc3 | 4321720 | 4574240 | 0,9447952 |
| Trol/5'-AZA1 | 4054280 | 4361288 | 0,92960612 |
| Trol/5'-AZA2 | 4187768 | 4421192 | 0,94720338 |
| Trol/5'-AZA3 | 4916568 | 4700944 | 1,04586823 |
