# Supplementary material for: Editorial Note: Iron-Ascorbate-Mediated Lipid Peroxidation Causes Epigenetic Changes in the Antioxidant Defense in Intestinal Epithelial Cells: Impact on Inflammation
Source: PLoS One. 2023 Jul 31;18(7):e0289575. doi: 10.1371/journal.pone.0289575 (PMC10389698; doi:10.1371/journal.pone.0289575)
Supplement: S2 File — (PDF) [file pone.0289575.s002.pdf]

Responses to the AE's comments
(https://doi.org/10.1371/journal.pone.0063456)

1. In Fig. 5A (published article), the Fe/Asc treatment induced SOD2 activity to >200U/mg protein compared to control (which may be less than 10U/mg protein based on the results in Fig. 5D, also in the published article). However, in Fig. 5D, Fe/Asc plus 5-AZA treatment brought down the SOD2 activity to the base line (control) level. As 5-AZA is a demethylation agent, Fe/Asc plus 5-AZA treatment would cause the SOD2 level at the same or higher than that by Fe/Asc treatment (>200U/mg protein in Fig. 5A).

About ten years ago, the objective of this work was to better understand the mechanisms of oxidative stress, very often accompanied by inflammatory processes, in the gastrointestinal tract. The importance of this exercise was to prevent or at least reduce the numerous gastrointestinal pathologies caused by oxidative stress. The main idea was to put special emphasis on the implication of an epigenetic redox regulation at the gastrointestinal level. For this reason, we exposed intestinal Caco-2/15 cells to iron/ascorbate (Fe/Asc)-mediated oxidative stress, followed by analysis of oxidative defense and methylation of SOD2 and GPx2.

Our initial expectation was that the activity of endogenous antioxidant enzymes should be reduced by iron/ascorbate-mediated oxidative stress, and that this could involve the methylation process. In accordance with the Reviewer's statement, we expected that the exposure of Caco-2/15 cells to 5-AZA (as a demethylating agent) would restore the expression and activity of both enzymes. First, the results showed that the Caco-2/15 cell model (originally derived from colorectal adenocarcinoma) is equipped with the antioxidant machinery needed to defend from radical-induced damage. Second, a different pattern characterized the activity and gene expression of the two enzymes tested in response to Fe/Asc: an increase for SOD2, and a decrease for GPx2. Third, the methylation was inverse: low for SOD2 and high for GPx2.

Examples with the same tendencies, especially emphasizing increased SOD2 activity/expression in correlation with positive DNA methylation, during the phase of oxidative stress, have been mentioned in the literature. In brief, while radical species generally decrease GPx, there are several conditions and models where oxidative stress stimulates SOD activity and/or expression. For example, the cigarette smoke-induced oxidative stress, as a major cause of ovarian injury (*Hum. Reprod. Update.* 2010;17:76–95; *Toxicol. Appl. Pharmacol. 2012;260:70–80*; *Zygote.* ***2017***;*25:296–303*), down-regulated 24 genes (including catalase and GPx) but caused an up-regulation of SOD2 and IL-6 (*Int J Mol Sci.* ***2021*** *Dec; 22(23): 13147*). Unfortunately, in this study, only the promoter of IL-6 was analyzed by pyrosequencing, which disclosed its hypomethylation. On the other hand, the administration of benzo[a]pyrene to Tegillarca granosa aroused oxidative stress (MDA) along with an increase of superoxide dismutase, concomitantly with global DNA methylation (*Sci Rep.* ***2021****; 11: 18744*). In this specific case, positive correlations were observed between SOD and oxidative DNA damage reflected by 8-OHdG. Another work underlines that animals exposed to pollution air particles resulted in

oxidative stress with significant increased SOD activities and DNA lesions, as well as decreased levels of the epigenetic mark 5-hydroxymethylcytosine in lungs (*Part Fibre Toxicol.* ***2018****; 15: 40*). In a different model, administration of arsenic caused oxidative stress and concomitantly an elevation of superoxide dismutase, along with DNA hypomethylation (*Environ Mol Mutagen. 2007 Oct;48(8):658-65*).
Overall, these various examples cited, in different models and experimental conditions, support our results concerning the profile of SOD2 in relation to the magnitude of DNA methylation.

As expected, the addition of 5-AZA to Caco-2/15 cells decreased the methylation process for GPx2 and restored its activity to normal levels. *However, the underlying mechanisms for the decay of SOD2 by 5-AZA to normal values (in the presence of Fe/Asc) are still unknown*, especially since the transcriptional and post-translational regulation of SOD2 is exceedingly complex. In addition, 5-AZA plays a role in regulating diverse transcription factors controlling cellular (HIF-1, SP1, SP3, NRF2, NF-kB, AHR) homeostasis and influencing SOD2 expression (*Bioengineered. 2021 Dec;12(1):2299-2310; Free Radic Biol Med. 2020 Feb 1;147:102-113; Carcinogenesis. 2013 Nov;34(11):2655-63; Mol Cancer Res. 2008 Dec;6(12):1881-93*). To give just one example related to iron used in our experiments to cause oxidative stress, we can mention the effects of 5-AZA on c-Myc. In fact, 5-AZA has been shown to activate transferrin receptor 1, ferritin, ferrochelatase and iron regulatory protein 2 (*Mol. Cell. Biol. 26, 2373–2386, 2006*; *Cancer Res.* 70, 6238–6246, 2010; *Science* 283, 676–679, 1999; *J Biol Chem. 2011 Oct 28; 286(43): 37196–37206*), all proteins involved in the transport, storage and metabolism of iron, which may have interfered with iron/ascorbate-mediated oxidative stress in our experiments.

These particular points from the scientific literature indicate that promoter demethylation may not be the only or the major mechanism by which 5-AZA affects gene expression. Regulation of gene expression by 5-AZA can occur in several ways, including some that are independent of DNA demethylation. Furthermore, the different mentioned studies suggest that the effect of 5-AZA on gene expression is highly context-dependent and can differ for the same gene under different environmental settings.

2. In Fig. 7A, the Fe/Asc treatment induced decreased methylation in the SOD2 promoter compared to control (consistent with increased SOD2 activity(protein) in Fig. 5A). However, in Fig. 7C, Fe/Asc plus 5-AZA treatment caused the SOD2 promoter methylation back to the base line (control) level. As 5-AZA is a demethylation agent, Fe/Asc plus 5-AZA treatment would cause demethylation further (more or less) in the SOD2 promoter, resulting in the same or lower methylation status (compared to control) than Fe/Asc treatment.

This remark of the Reviewer is quite understandable since we also expected a greater degree of SOD2 promoter demethylation in the presence of Fe/Asc and 5-AZA. It may be that the two components (Fe/Asc & 5-AZA) act somewhat differently with respect to the methylation process. While it is established that 5-AZA decreases 5-methylcytosine levels by inhibiting the enzymes responsible for DNA methylation, it is not quite the same with

iron. This essential trace metal is not only associated with the redox system, but can also promote the activity of DNA methyltransferases. For example, iron may increase DNA methyltransferases as evidenced by iron-specific chelators, which downregulated DNA methyltransferases in liver cells (*Heliyon. 2020 Oct; 6(10): e05059*). Apparently, iron-deficient conditions lowered cellular DNA methyltransferases levels by increasing ROS production, leading to decreased levels of protein methylation. Similarly, dietary iron-deficient rats showed decreased DNA methyltransferases expression and protein methylation (*Heliyon. 2020 Oct; 6(10): e05059*). *Therefore, a possible explanation could be that the combination of iron/Asc (provided for oxidative stress induction but with a methylating influence) and 5-AZA (as a demethylating agent) has contributed to the findings. In addition, as previously discussed, cellular functional changes caused by 5-AZA (proliferation, metabolism, epigenetics) and iron metabolism alter the methylation response.*

3. Fig. 3D showing the 5-AZA treatment decreased the NF-kB protein to control level is not supported by other results. Why and how did the 5-AZA treatment bring the increased NFkB (Fig. 3A) down to the control level?

Our results are well supported by the scientific literature. In different cells and conditions, the exposure to 5-AZA resulted in inflammation alleviation via the downregulation of NF-κB. In renal cells and B cells, 5-AZA suppressed phosphorylation and activity of NF-κB (*Int J Clin Exp Pathol.* ***2015****; 8(9): 11140–11148; Cell Signal.* ***2015*** *May;27(5):969-77*). Decreased expressions of NF-kB were noted in response to 5-AZA in esophageal squamous cells (*Biomed Pharmacother. 2014 Jul;68(6):745-50*). Moreover, in mice with inflammation and sepsis, 5-AZA reduces NF-κB activation in mononuclear cells (Front Immunol. **2020** Jul 2;11:1360). Furthermore, 5-AZA prevents arsenite-*induced hypermethylation and blocks arsenite-induced activation of the* Ras/NF-κB signal pathway (*Toxicol Lett. 2014 Jun 5;227(2):91-8*). In MCL *cell lines, 5-AZA downregulated nuclear factor (NF)-κB-p65 (Br J Haematol. 2013 May;161(3):348-56).*

4. The same issue on SOD2 expression (% of control) between Fe/Asc (Fig.6A) and Fe/Asc plus 5-AZA in both the original and repeated data. The much higher SOD2 by Fe/Asc than Fe/Asc plus 5-AZA (Fig. 6A and 6C) does not look consistent with the methylation status in Fig. 7A and 7C.

Please see the response to comment 1, which also applies to this comment

5. The results section Reactivation of SOD2 and GPx2 expression after treatment with 5-Aza-2'-deoxycytidine states "As shown in Figures 5D and 5E, the activity of both enzymes was re-established at the same level of control cells after treatment with 5-AZA. In parallel, 5-AZA restored gene expression (Figures 6C and 6D) and the DNA methylation (Figures 7C and 7D) of these antioxidants and counteracted all the Fe/Asc effects." The data suggest that 5-AZA restored gene expression in Fig. 6D (GPx) but not in Fig. 6C (SOD2) because SOD2 expression was already induced by Fe/Asc (Fig. 6A).

We have dealt with this issue in the response of comment 1.

6. The Fe/Asc control is missing from the results in Figures 3D, 3E, 5D, 5E, 7C, and 7D.

Fe/Asc-induced oxidative stress (as observed in Figure 2) produced high levels of MDA (Fig 2), impaired antioxidant defense (SOD2 & GPx) (Fig 5), and increased inflammatory factors (IL-6, COX2, and NF-*κ*B) (Fig 3 and 4) compared with control and Trolox. Our expectation was that if 5-AZA reduced the values of these variables to those of control and Trolox, this was sufficient to document the effect of 5-AZA-mediated demethylation, and it was therefore not relevant to add Fe/Asc as a control.

7. The quantitative repeat experiment data provided for Figure 3E appear different from the results presented in the published article. Please comment on this difference and how it affects the published results, especially the last statement in the results section: "*Moreover, it resulted in the substantial reduction of MDA, but without a complete neutralization of lipid peroxidation (Figure 2), the prevention of NF-κB increase (Figure 3D) and IκB decrease (Figure 3E) protein expression as well as in normalization of NF-κB/IκB ratio (Figure 3F), IL-6 and COX-2 protein expression (Figures 4C and 4D).*"

In the published article: Caco-2/15 cells exposed to Fe/Asc displayed a high NF-κB signal (Fig 3A) along with a low level of IκB protein expression (Fig 3B), which culminated in a high ratio of NF-κB/IκB (Fig 3C), which underlies NF-κB activation and pro-inflammatory function.
In contrast, the presence of 5-AZA suppressed both the Fe/Asc-mediated NF-κB increase (Fig 3D) and Fe/Asc-mediated I-κB decrease (Fig 3E) without no significant change in the NF- κB/IκB ratio (Fig 3F), indicating no NF-κB activation and pro-inflammatory function.
In the recently repeat experiment data : (Figures 3D-3E-3F). A similar pattern was obtained. Indeed, no statistically significant differences were noticed in NF- κB (3D), I-κB (3E) and NF-κB/IκB ratio (3F), indicating no NF-κB activation and pro-inflammatory function. Therefore, **the message delivered in both versions is exactly the same**. Of special note, in both versions (published article & repeat experiment data), no statistically significant differences were recorded in Figure 3E, **which does not affect the message of the published article**.
